# Study of interaction of antimutagenic 1,4-dihydropyridine AV-153-Na with DNA-damaging molecules and its impact on DNA repair activity

**DOI:** 10.7717/peerj.4609

**Published:** 2018-04-25

**Authors:** Elina Leonova, Evita Rostoka, Sylvie Sauvaigo, Larisa Baumane, Turs Selga, Nikolajs Sjakste

**Affiliations:** 1 Faculty of Medicine, University of Latvia, Riga, Latvia; 2 Latvian Institute of Organic Synthesis, Riga, Latvia; 3 LXRepair, Grenoble, France

**Keywords:** 1,4-dihydropyridines, AV-153-Na, DNA repair

## Abstract

**Background:**

1,4-dihydropyridines (1,4-DHP) possesses important biochemical and pharmacological properties, including antioxidant and antimutagenic activities. It was shown that the antimutagenic 1,4-dihydropyridine AV-153-Na interacts with DNA. The aim of the current study was to test the capability of the compound to scavenge peroxynitrite and hydroxyl radical, to test intracellular distribution of the compound, and to assess the ability of the compound to modify the activity of DNA repair enzymes and to protect the DNA in living cells against peroxynitrite-induced damage.

**Methods:**

Peroxynitrite decomposition was assayed by UV spectroscopy, hydroxyl radical scavenging—by EPR spectroscopy. DNA breakage was determined by the “comet method”, activity of DNA repair enzymes—using Glyco-SPOT and ExSy-SPOT assays. Intracellular distribution of the compound was studied by laser confocal scanning fluorescence microscopy. Fluorescence spectroscopy titration and circular dichroism spectroscopy were used to study interactions of the compound with human serum albumin.

**Results:**

Some ability to scavenge hydroxyl radical by AV-153-Na was detected by the EPR method, but it turned out to be incapable of reacting chemically with peroxynitrite. However, AV-153-Na effectively decreased DNA damage produced by peroxynitrite in cultured HeLa cells. The Glyco-SPOT test essentially revealed an inhibition by AV-153-Na of the enzymes involved thymine glycol repair. Results with ExSy-SPOT chip indicate that AV-153-Na significantly stimulates excision/synthesis repair of 8-oxoguanine (8-oxoG), abasic sites (AP sites) and alkylated bases. Laser confocal scanning fluorescence microscopy demonstrated that within the cells AV-153-Na was found mostly in the cytoplasm; however, a stain in nucleolus was also detected. Binding to cytoplasmic structures might occur due to high affinity of the compound to proteins revealed by spectroscopical methods.

**Discussion:**

Activation of DNA repair enzymes after binding to DNA appears to be the basis for the antimutagenic effects of AV-153-Na.

## Introduction

Synthetic derivatives of 1,4-dihydropyridine (1,4-DHPs) possess important biochemical and pharmacological properties. They show modulating activity on cardiovascular and neuronal processes as well as anticancer, genoprotective and radioprotective effects. In the present investigation we have focused our attention on a representative of the 1,4-DHP derivatives, which is considered to be “unusual”. These compounds are water-soluble molecules without the activity of blockers of calcium channels or with a very weak blocking activity. 1,4-DHPs of this group manifest different biological activities, including genome-protecting effects; for example, glutapyrone is an antineoplastic and anticlastogenic agent, antimutagen and enhancer of DNA repair (Goncharova et al., 2001; Kuzhir, Dalivelia & Savina, 1999; Vartanian et al., 2004). Our interests were focused on the compound AV-153-Na possessing antimutagenic activity and being an enhancer of DNA repair (Ryabokon, Cieslar-Pobuda & Rzeszowska-Wolny, 2009; Ryabokon et al., 2008; Ryabokon et al., 2005; Ryabokon et al., 2009). Recently, we have revealed the DNA binding capacity of this compound (Buraka et al., 2014). The aim of the current study was to test the DNA-protective capability of the compound in formerly unstudied systems, to test the ability of the compound to scavenge peroxynitrite and hydroxyl radical, and to assess the ability of the compound to modify the activity of DNA repair enzymes. To achieve these goals, the study was designed as follows. Firstly we have evaluated possibility of direct interaction of the compound with DNA-damaging agents, hydroxyl radical and peroxynitrite *in vitro*, in order to reveal the role of direct chemical interactions in antimutagenic activity of AV-153-Na. Further, possible impact of the AV-153-Na on the dynamics of DNA breakage in living cells, and activity of DNA repair enzymes was studied by means of single cell electrophoresis and functional repair assays (Glyco-SPOT and ExSy-SPOT assays).

## Materials and Methods

### Chemicals

**Figure 1:**
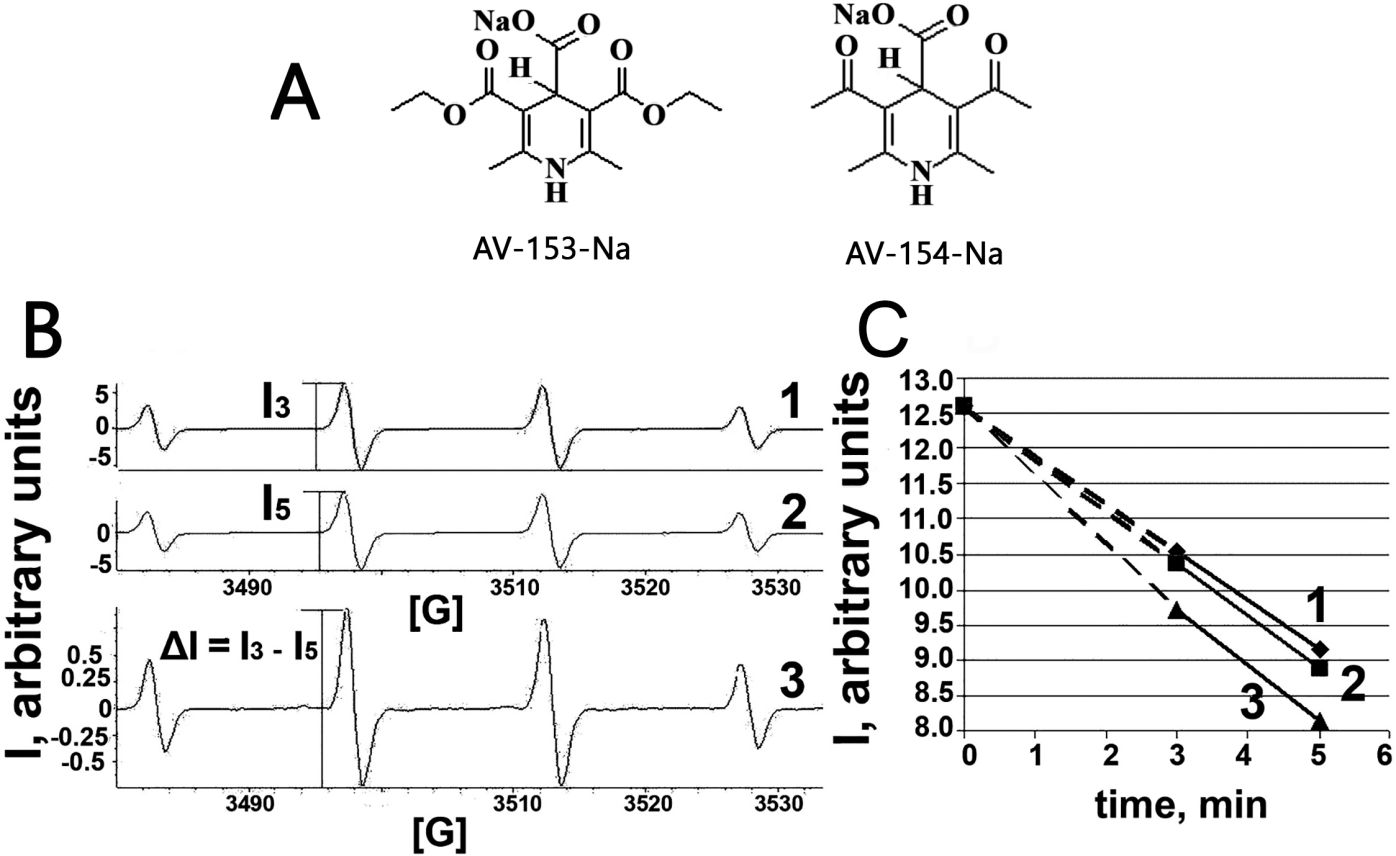
Electron paramagnetic resonance spectroscopy. (A) Chemical structures of AV-153 –Na and AV-154. (B) EPR spectra of DMPO-OH radicals generated in Fenton reaction in presence of DMPO. 1—EPR spectra of DMPO-OH radicals 3 min after mixing the components for Fenton reaction. 2—EPR spectra of DMPO-OH radicals 5 min after mixing the components for Fenton reaction. I3 and I5—intensities of EPR signals used for quantification of DMPO-OH radicals in corresponding time. 3—difference between 3 min and 5 min spectra indicating decrease of the signal intensity and lack of generation of other radicals. (C)—time course of decrease of intensity of DMPO-OH radical spectra. 1—control mixture; 2—in presence of AV-154-Na; 3—in presence of AV-153-Na.

AV-153-Na and AV-154-Na were synthesized in the Laboratory of Membrane Active Compounds at the Latvian Institute of Organic Synthesis. Structures of the AV-153-Na and AV-154-Na are given in Fig. 1; the synthesis of the compounds was performed essentially as described (Dubur & Uldrikis, 1969). Tris base, ferrous sulphate, 5,5-dimethylpyrroline-*N*-oxide (DMPO), Triton-X-100, human serum albumin (HSA), Na_2_EDTA, LiCl, NaCl, CaCl_2_ and other inorganic salts were purchased from Sigma-Aldrich (Taufkirchen, Germany). Peroxynitrite was synthesized as described by Robinson & Beckman (2005).

### Cell culture

HeLa cells (Biomedical Research and Study Centre, Riga, Latvia) were grown in DMEM + GlutamaxTM–I, F-12 Nut-Mix (1×) (Sigma-Aldrich, Taufkirchen, Germany) + 10% fetal bovine serum (Sigma-Aldrich, USA), at 37 °C in a humidified atmosphere containing 5% CO_2_.

### Fenton reaction—ESR measurements

For the Fenton reaction (Fe^2+^ + H_2_O_2_ → Fe^3+^ + ⋅OH + OH^−^), 80 µl of reaction mixture containing 250 µM ferrous sulphate, 250 µM H_2_O_2_, 80 mM spin trap DMPO, and 1 mM of 1,4 DHP was transferred to a micro pipettes tube for measurement of the electron spin resonance (ESR) spectra of DMPO-OH radicals. ESR spectra of the spin trap and radical complex were recorded at room temperature using an EMX-plus EPR spectrometer (Bruker, Germany). The EPR instrumental settings for field scan were as follows: field sweep—100G; microwave frequency—9.84 GHz; microwave power—15.9 mW; modulation amplitude—1 G; conversion time—163 ms; time constant—327 ms; sweep time –83 s; receiver gain–1⋅10^4^; resolution—512 points for 1 scan.

### The single cell electrophoresis (comet assay)

The single cell electrophoresis (comet assay) was performed as described previously (Leonova et al., 2016)

### UV/VIS spectroscopic measurement of peroxynitrite decomposition

The rate of peroxynitrite (0.38 mM) decomposition in the presence or in the absence of the 1,4-DHP (0.16 mM) was followed at 302 nm (absorbance peak for the peroxynitrite anionic form) in 10 mM Tris pH 10 buffer on Perkin Elmer Lambda 25 UV/VIS spectrophotometer (Carballal, Bartesaghi & Radi, 2014). The average rate of reactions were calculated according to the formula *V* =  ± ((*C*_2_ − *C*_1_)∕(*t*_2_ − *t*_1_)) =  ± (Δ*C*∕Δ*t*), where *C*_1_ was the concentration of peroxynitrite in the beginning of reaction, and C_2_ the concentration of peroxynitrite at the end of the reaction; Δ*t*: 20 min.

### Circular dichroism spectroscopy

CD spectra were recorded on a Chirascan CS/3D spectrometer (Applied Photophysics, Surrey, UK); CD spectra of HSA in the absence or in the presence of AV-153-Na salts were recorded in PBS buffer, pH 7.4 in a range of 200–260 nm. A 300 nM HSA solution was titrated with AV-153-Na (1 µM at each step) in a quartz cell of 10 mm path-length at room temperature. The parameters for the spectra were as follows: scan rate—200 nm min^−1^, averaging time— 0.125 s, bandwidth—1 nm; one recorded spectrum is the average of four scans.

### Fluorescence spectroscopic measurements

Spectrofluorimetric analyses were performed on a Fluoromax-3 (Horiba JOBIN YVON, Shanghai, China), essentially as described (Osina et al., 2017).

### Cell treatment and nuclear extract preparation for repair reactions

HeLa cells were incubated with 50 nM of AV-153-Na for 3, 12 or 24 h, washed with PBS, trypsinized, suspended in PBS and pelleted by low-speed centrifugation. Cell pellets were incubated in ice for 20 min in 1.25 mL of ice-cold buffer A (10 mM HEPES pH 7.9, 1.5 mM MgCl_2_, 10 mM KCl, 0.01% Triton X-100, 0.5 mM DTT, 0.5 mM PMSF) and vortexed for 30 s. After centrifugation for 5 min at 5,000 rpm at 4 °C, the nuclei were suspended in 31.25 µL of ice-cold buffer B (10 mM HEPES pH 7.9, 1.5 mM MgCl_2_, 400 mM KCl, 0.2 mM EDTA, 25% glycerol, 0.5 mM DTT, antiproteases (Complete-mini, Roche, Meylan, France) and 0.5 mM PMSF). The nuclear membrane lysis was completed by incubation for 20 min on ice, followed by two cycles of freezing-thawing at −80 °C and 4 °C respectively. Debris was eliminated by centrifugation for 10 min at 13,000 rpm at 4 °C. The supernatant was stored frozen in 10 µl aliquots at −80 °C. Protein content was determined using the BCA kit (Interchim, Montluçon, France). Typical protein content was 0.8 mg/mL.

### Assays of the activity of DNA repair enzymes

The impact of the tested compounds on activity of glycosylases/AP endonucleases belonging to Base Excision Repair and on Excision/Synthesis Repair activities was performed using Glyco-SPOT (Pons et al., 2010) and ExSy-SPOT assays (Forestier et al., 2012), respectively (LXRepair, Grenoble, France). These assays allowed quantifying different DNA repair activities from extracts prepared from treated and non-treated cells.

The former chip, which is a multiplex, on-support, oligonucleotide (ODN) cleavage assay, reveals excision activities against 8-oxoguanine paired with C (8-oxoG-C), A paired with 8oxoguanine (A-8oxoG), ethenoadenine (EthA-T), thymine glycol (Tg-A), uracil (paired either with G or A (U-G and U-A, respectively)), hypoxanthine (Hx-T), and abasic sites (THF-A). Cleavage of the lesions by the enzymes contained in the extracts released the fluorescence attached to the lesion containing ODNs.

Repair reactions were conducted for 1 h at 37 °C with 15 µg/mL of protein in 80 µL of excision buffer (10 mM Hepes/KOH pH 7.8, 80 mM KCl, 1 mM EGTA, 0.1 mM ZnCl_2_, 1 mM DTT, 0.5 mg/mL BSA). After 3 washes, 5 min at room temperature in PBS containing 0.2 M NaCl and 0.1% Tween 20, the spots fluorescence was quantified using the Innoscan scanner from Innopsys (Toulouse, France). Each extract was run in duplicate. The results between the replicates (4 spot fluorescence) were normalized using the NormalizeIt software as described by Millau et al. (2008).

Wells incubated with the excision buffer served as reference (100% fluorescence) to calculate the lesions percentage of cleavage in the wells incubated with the extracts. Non-specific cleavage of the control ODN (Lesion_Free ODN) was also taken into account to calculate the percentage of excision of each lesion using the following formula: (100 ×(1−percentage of fluorescence of Lesion_ODN/percentage of fluorescence of Lesion_Free ODN)).

The ExSy-SPOT assay quantified Excision/Synthesis Repair of 8-oxoguanine (8oxoG), alkylated bases (AlkB) and abasic sites (AbaS), incorporated into different supercoiled plasmid DNA. The principle of the methods is described by Millau et al. (2008). Extracts (0.1 mg/mL) incubated on the biochip where the different plasmid preparations were immobilized at specific sites for 3 h at 30 °C in reaction buffer (40 mM Hepes KOH pH 7.8, 7 mM MgCl2, 0.5 mM DTT, 0.25 µM dATP, 0.25 µM dTTP, 0.25 µM dGTP, 3.4% glycerol, 12.5 mM phosphocreatine (Sigma, Taufkirche, Germany), 2 mM EDTA, 50 µg/mL creatine phosphokinase, 0.1 mg/mL BSA) containing 1 mM ATP (Amersham, England) and 1.25 µM dCTP-Cy3. After washing for 3 × 5 min in H_2_O (MilliQ), the total fluorescence intensity of each spot was quantified using the Innoscan scanner from Innopsys (Toulouse, France). Each extract was run in duplicate and data were normalized using the NormalizeIt software as described by Millau et al. (2008). Results were expressed as Fluorescence Intensity (FI).

### Laser confocal scanning microscopy

For imaging, the HeLa cells were seeded in 4 well Nunc Lab-Tek Permanox Chamber slide (Thermo Scientific Nunc, Pittsburg, PA, USA) and cultivated for 24 h as described above. Subsequently, the cells were washed twice with PBS for 5 min and then incubated with 1 mM AV-153-Na in PBS for 16 h in a CO_2_ incubator at 37 °C and 5% CO_2_. After incubation, the cells were washed with PBS for 5 min and fixed in 70% ethanol for 0.5 h at room temperature. Slides were rinsed with PBS for 5 min and counterstain chromatin was dyed with 15 µM propidium iodide (PI) in PBS for 0.5 h, then washed twice with PBS for 5 min. Slides were analyzed using a Leica DM RA-2 microscope equipment with a TCS-SL confocal scanning head (Leica Microsystems, Bannockburn, IL, USA). Images were collected with a Leica 40× HCX PL Fluator objective (NA = 0.75) and 100× HCX PIAPO oil immersion objective (NA = 1.40). AV-153-Na and propidium iodide were excited with a 488 nm band from a four-line argon ion laser. AV-153-Na fluorescence was detected between 510 and 560 nm, propidium iodide fluorescence was detected between 600 and 650 nm. Cell shapes were controlled with reflected light 475–505 nm. Cells were scanned along the *Z*-axis with a step size—0.5 µm.

### Statistical analysis

The values of DNA damage assayed by single gel electrophoresis are represented as the mean ± standard error of the mean (SEM). The data were subjected to the one-way analysis of variance (ANOVA), followed by the Tukey multiple comparisons test and the data were considered as significant at *p* < 0.05.

## Results

### Study of chemical interactions of AV-153-Na with DNA-damaging agents

Taking into account capability of the AV-153-Na to decrease DNA damage produced by hydrogen peroxide (Ryabokon et al., 2008; Ryabokon et al., 2005) we have tested the ability of the AV-153-Na to scavenge free radicals. The OH radical produced in Fenton reaction, was tested by the ESR method. We have tested AV-153-Na at 1,000 µM concentration. The signals of the second component of the EPR spectra were measured on the 3rd min (I_3_) and the 5th min (I_5_) and the difference between I_3_ and I_5_ was calculated (Fig. 1). Scavengers of OH radicals should increase the difference between I_3_ and I_5_. Representative kinetics of the decrease of EPR signal intensity is shown in Fig. 1C. The impact of AV-153-Na on the signal intensity is minimal, AV-154-Na does not produce any effect at all. Thus these 1,4-DHP derivatives are very weak radical scavengers.

**Figure 2:**
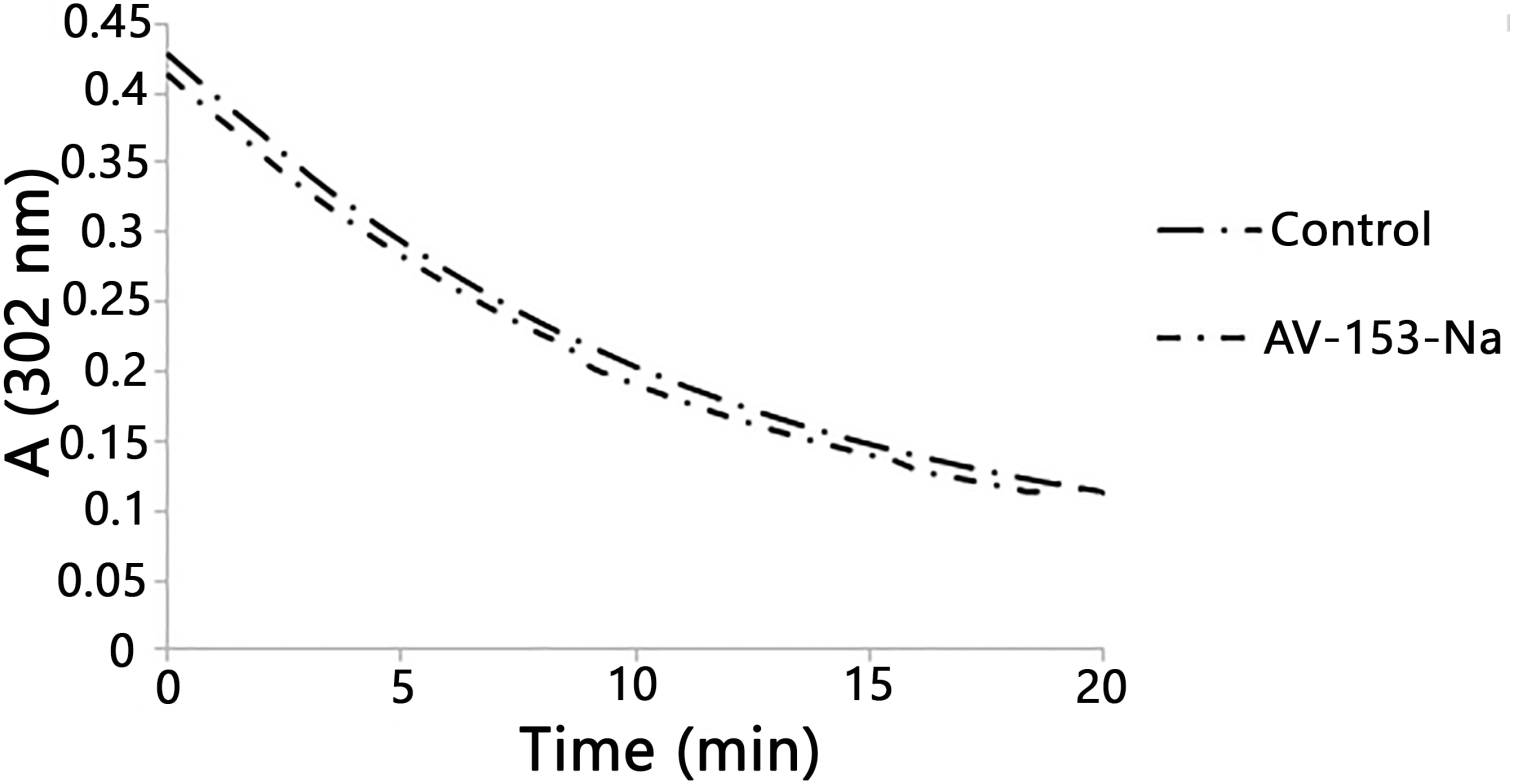
Reaction with peroxynitrite assay. Curves demonstrate decomposion of peroxynitrite (0.38 mM) in the presence of AV-153-Na added up to 0.16 mM at pH 10. The 1,4-DHP was added also to the control cuvette.

Published data indicate the ability of some 1,4-DHP to scavenge peroxynitrite chemically (Lopez-Alarcon et al., 2004). We also tested the ability of the AV-153-Na salts to degrade peroxynitrite chemically by studying the kinetics of decomposition of peroxynitrite in the presence of DHP followed by means of spectrophotometry. The curves are presented in Fig. 2. The average rate of spontaneous decomposition of peroxynitrite at concentration 0.38 mM was 0.0157 µmol/µl min. AV-153-Na did not affect the time of decomposition of peroxynitrite; it remained 0.0157 µmol/µl min.

### Intracellular localization of AV-153-Na and protein binding

**Figure 3:**
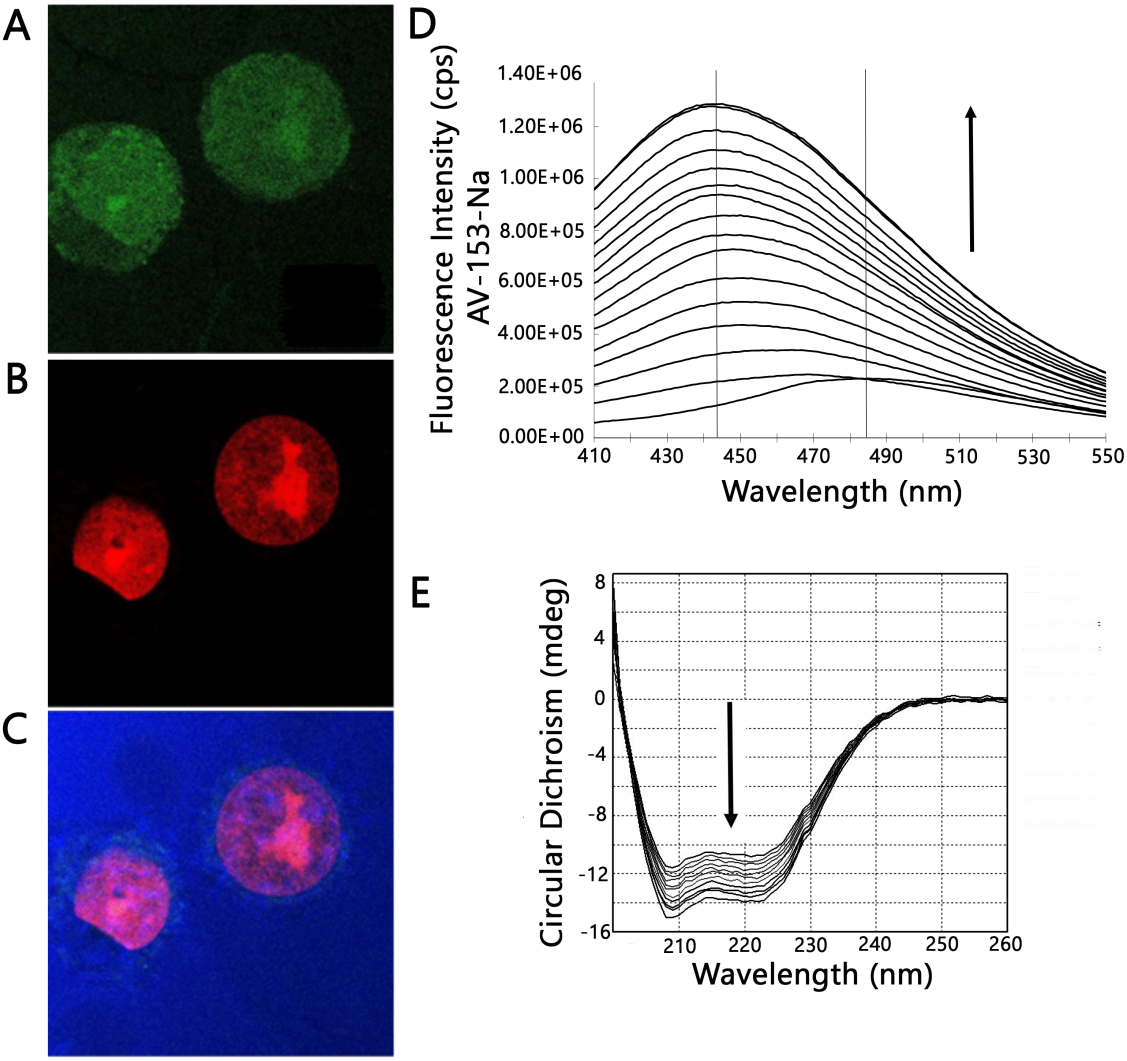
Confocal microscopy and protein binding assay. (A–C) images of HeLa cells obtained by laser scanning confocal microscopy. Cells were treated with AV-153-Na, DNA was stained with propidium iodide (PI). Light blue—reflected light; (A) green—distribution of AV-153-Na; (B) red—propidium iodide; the overlay image (C) of all channels. Pictures of the optical section were taken 3 µm from the cell surface. All the scale bars are in 7.5 µm size. (D) Fluorescence titration of AV-153-Na with human serum albumin (5 µM each time). (E) Circular dichroism spectra of HSA in absence and presence of AV-153-Na. AV-153-Na concentration was increased by 1 µM at each step up to 12 µM. HSA concentration was 300 nM.

As the capability of AV-153-Na to bind DNA *in vivo* was proven previously (Buraka et al., 2014), its antimutagenic effect could not be attributed to chemical interactions with DNA-damaging agents (see above) we needed evidence whether the compound could reach cell nucleus in the cells where it could interact with DNA. We have tried to answer this question by profiting of the intrinsic fluorescence of the compound with the aid of laser confocal scanning microscopy. Results are presented in Figs. 3A and 3C. In most cells AV-153-Na heavily stains the cytoplasm; however, some fluorescence is visible also in the nucleus, mainly in the nucleolus. Staining of cytoplasmic structures raised the question about capability of the compound to bind proteins. Fluorescence titration of AV-153-Na with human serum albumin gave a positive answer to the question - fluorescence of the compound increased in the presence of HSA. Results of CD confirmed the ability to bind a protein (Figs. 3D and 3E). The addition of the compound to the HSA solution decreased CD signals in both minimal bands (208 and 222 nm) in the presence of AV-153-Na, which suggests binding with HSA, causing protein conformational changes due to a slight protein unfolding (Wang et al., 2008).

### Impact of AV-153-Na on DNA damage and DNA repair systems

**Figure 4:**
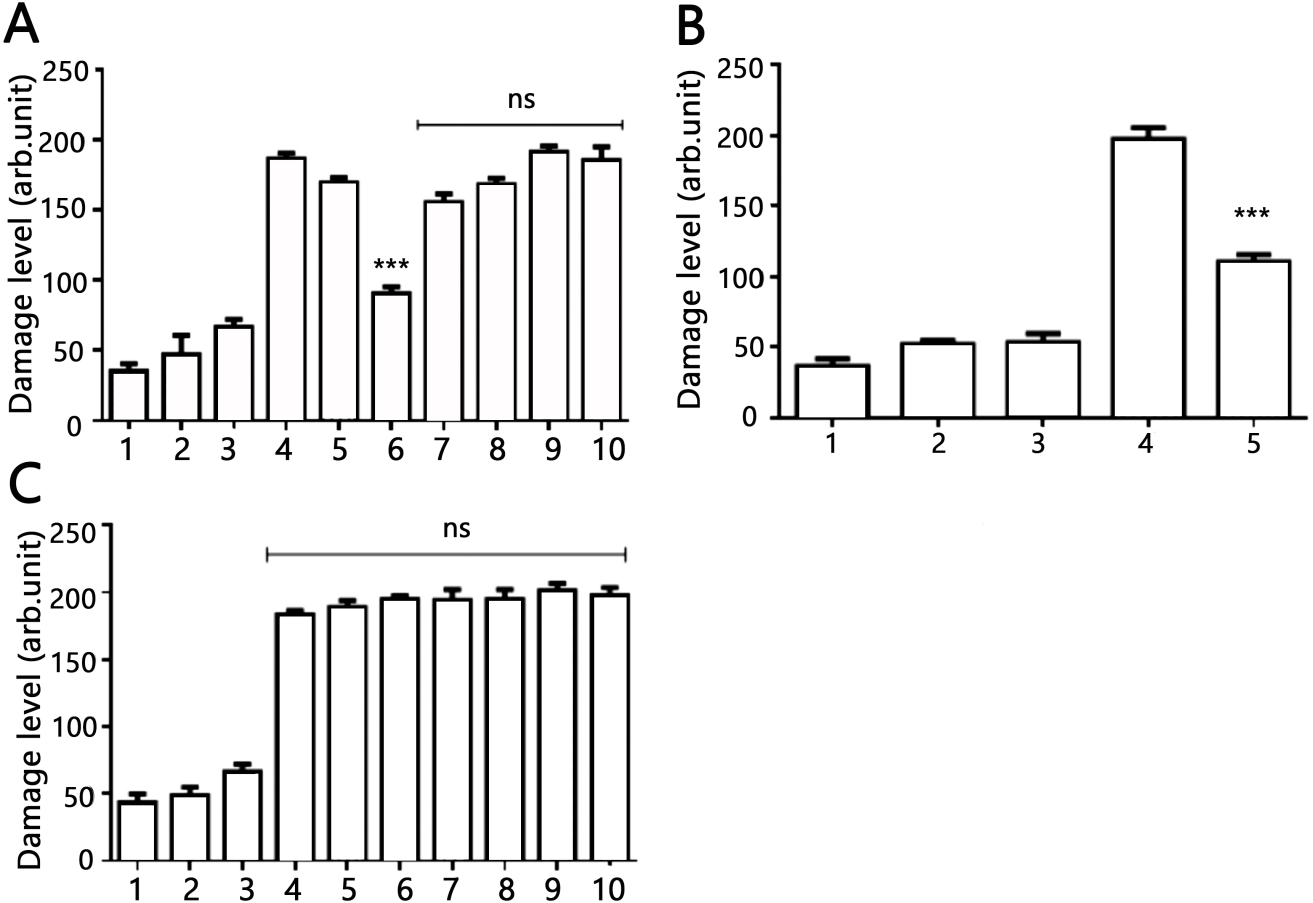
Effects of AV-153-Na and AV-154-Na against peroxynitrite caused DNA damage in HeLa cell line tested by comet assay. (A) AV-153-Na. 1, control (intact cells); 2, vehicle control (60 µM of NaOH); 3, peroxynitrite (200 µM); 4, incubation with the tested 1,4-DHP (100 nM, 45 min), 5, pre-incubation with 10 nm of 1,4-DHP (45 min) and treatment with peroxynitrite; 6, pre-incubation with 50 nm of 1,4-DHP (45 min) and treatment with peroxynitrite; 7, pre-incubation with 100 nm of 1,4-DHP (45 min) and treatment with peroxynitrite; 8, simultaneous treatment with 1,4-DHP (10 nm) and peroxynitrite; 9, simultaneous treatment with 1,4-DHP (50 nM) and peroxynitrite; 10, simultaneous treatment with 1,4-DHP (100 nM) and peroxynitrite; (B) AV-153-Na. 1, control (intact cells); 2, incubation with 50 nM of AV-153-Na for 3 h; 3, vehicle control (60 µM of NaOH; 3 h); 4, peroxynitrite (200 µM); 5, pre-incubation with 50 nm of AV-153-Na (3 h) and treatment with peroxynitrite; (C) AV-154-Na, all designations are as in A. ∗∗∗ - *p* < 0.001 versus peroxynitrite group, ns, not significant.

The DNA-protecting action of AV-153 salts against peroxynitrite-induced damage was tested in living cells. Results of the comet assay experiments performed on HeLa cell treated with peroxynitrite alone or in the presence of AV-153-Na are presented in Fig. 4. Treatment with peroxynitrite drastically increased the levels of DNA damage. AV-153-Na reduced the extent of DNA damage produced by peroxynitrite. Pre-incubation with the compound at concentrations 50 nM for 45 min appeared to produce significant effects (Fig. 4A). When administered simultaneously with peroxynitrite, the compound produced a much weaker protective effect. AV-154-Na, which does not bind DNA (E Leonova et al., 2018, unpublished data), did not protect the cells against peroxynitrite (Fig. 4C).

Effects of AV-153-Na on the activity of DNA repair enzymes were tested using Glyco-SPOT and ExSy-SPOT assays. Longer pre-incubation times were chosen to reveal possible changes in protein expression.

**Figure 5:**
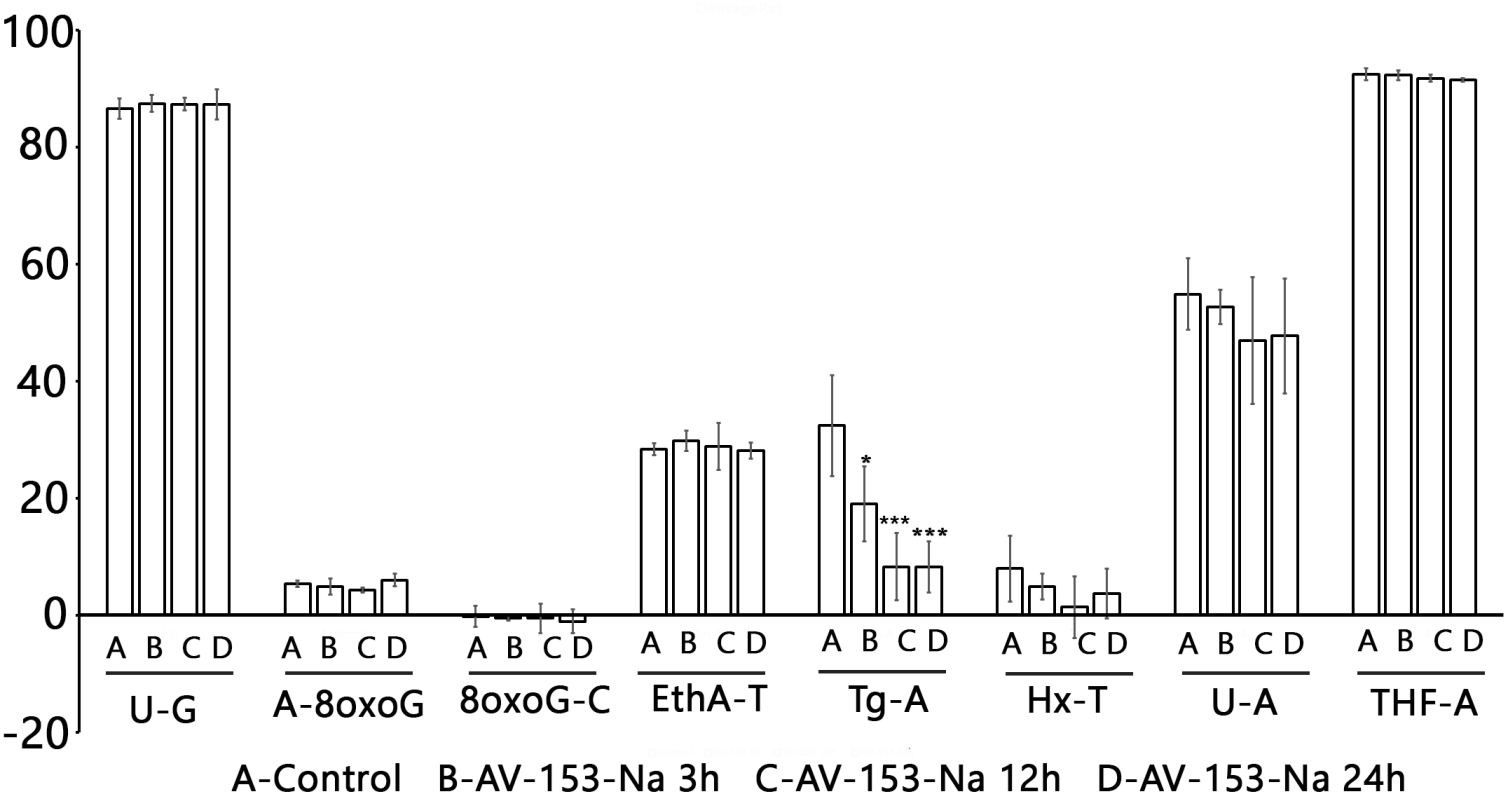
Effect of AV-153-Na on cellular Base Excision Repair activities (Glyco-SPOT assay). The test was run with 15 µg/ml of extract prepared from non-treated cells (Control) and cells treated for 3 h, 12 h and 24 h with AV-153-Na as described in Materials and Methods. Results are expressed as cleavage rate for each lesion. U-G and U-A : uracil paired either with G or with A; A-8oxoG: A paired with 8oxoguanine; 8oxoG-C: 8-oxoguanine paired with C; EthA-T ethenoadenine paired with T; Tg-A : thymine glycol paired with A; Hx-T : hypoxanthine paired with T; THF-A : abasic sites analogue paired with A. ^∗^*p* < 0.05, ^∗∗∗^*p* < 0.001 versus Tg-A control.

The Glyco-SPOT assay revealed a specific and significant decrease of Tg (thymine glycol) repair by AV-153-Na, which manifested itself when an extract with a higher concentration of protein (15 µg/ml) was used in the assay, and a trend for inhibition of enzymes involved in U-G and U-A repair (Fig. 5). Other glycosylases/AP endonucleases activities were not affected.

**Figure 6:**
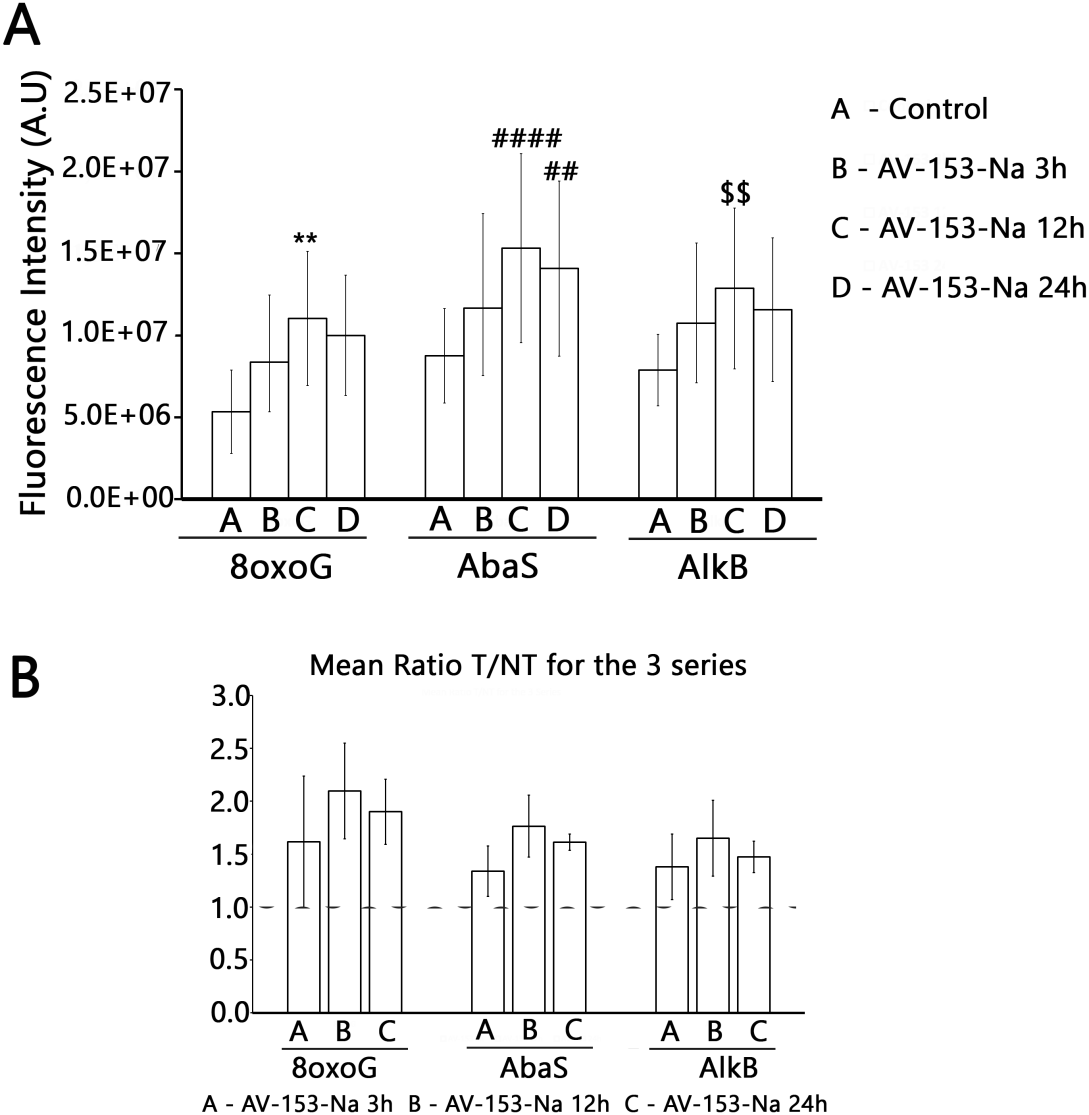
Effect of AV-153-Na on cellular Excision/Synthesis Repair (ExSy-SPOT assay) of major base lesions. The repair reaction was conducted with nuclear extracts prepared from non-treated cells and cells treated for 3 h, 12 h and 24 h with AV-153-Na (see Materials and Methods). For each lesion, we calculated the ratio of the fluorescence intensity obtained with the treated cells over the fluorescence intensity obtained with the control cells. The values >1 reflect an induction of the Excision/Synthesis Repair activities. ^∗∗^*p* < 0.01 versus 8oxoG control; ^####^*p* < 0.0001 versus AbaS control; ^∗^*p* < 0.01 versus AlkB control.

Results with ExSy-SPOT assay appear to be more interesting in this sense. AV-153-Na stimulates the excision/synthesis repair of lesions repaired by Base Excision Repair (8-oxoG, abasic sites and alkylated bases (Fig. 6)). As this stimulating effect is not detected with the Glyco-SPOT assay, it involves either the synthesis step of the repair process or alternative repair pathways able to handle oxidative lesions.

## Discussion

In order to test if the antimutagenic effects of the AV-153-Na are due to its capability to scavenge free radicals and peroxynitrite, we have studied these effects using *in vitro* systems, as some 1,4-DHP are able to scavenge different reactive oxygen and nitrogen species themselves; the reactions can be observed *in vitro* (Pacheco et al., 2013; Vijesh et al., 2011). Unexpectedly, it turned out that the compound does not react with peroxynitrite, and ability to scavenge hydroxyl radical turned out to be modest, thus the cause of the antimutagenic effect should have been sought inside the cells. We report evidence of possible binding of the AV-153-Na to cell nucleus. Thus the DNA-binding activity of the compound could be responsible for its antimutagenic activity.

However, binding to DNA is usually considered to be mutagenic, this might seem to be in contradiction with its reported antimutagenic and DNA-protecting activities of the compound (Goncharova et al., 2001; Ryabokon, Cieslar-Pobuda & Rzeszowska-Wolny, 2009; Ryabokon et al., 2008; Ryabokon et al., 2005; Ryabokon et al., 2009). In contradiction to this point of view, analysis of literature data reveals the coexistence of DNA-binding activity with antimutagenic effects. Natural polyphenols provide a good illustration of this statement: many of them effectively bind DNA through intercalation; however, most of these compounds are considered to be antimutagenic. The latter activity is attributed to antioxidant properties of this class of compounds (Janjua et al., 2009; Zhang et al., 2011). It appears that the DNA-damaging and DNA-protecting activities cohabitate in the molecules of flavonoids. Even better example is presented by widely used antidiabetic drug metformin. It protects the type 2 *diabetes mellitus* patients from cancer development (Safe, Nair & Karki, 2018), it protects DNA against breakage (Algire et al., 2012), it interferes with efficiency of the DNA repair (Lee et al., 2016) but it binds the DNA (Shahabadi & Heidari, 2012).

Data of comet assay when the AV-153-Na was tested for ability to modify level of DNA breakage in HeLa cells exposed to peroxynitrite provide more evidence for the role of the DNA binding in the mutagenic effect of the compound. Pre-incubation with AV-153-Na significantly decreased the DNA damage. Perhaps a higher efficiency of low concentrations of AV-153-Na reflects a shift of the equilibrium between DNA damage being a consequence of DNA-binding and DNA protection towards DNA protection. The necessity for pre-incubation and lower efficiency of simultaneous administration with peroxynitrite indicates that AV-153-Na induces some changes in the cells favouring protection of DNA or DNA repair, as the compound does not interact directly with the peroxynitrite. Moreover, the good DNA binder AV-153-Na was an effective DNA protecting agent, while AV-154-Na, which does not interact with DNA at all, did not protect it against peroxinitrite. It seems that data on the impact of AV-153-Na on the activity of the excision repair enzymes makes understanding of the mechanism of action of the compound possible. AV-153-Na activates enzymes involved in the excision repair pathway. The observed decrease in Tg removal produced by AV 153-Na apparently contradicts data about DNA-protecting effects of the compound. However, it should be taken into account that in mammals two bifunctional glycosylases, NTH1 and NEIL1, show overlapping activities aimed on the removal of Tg (Sampath, 2014). Our data do not permit us to determine which enzyme was inhibited. Although this finding cannot explain the DNA-protecting effects of AV-153-Na, it appears to be interesting.

The study further reveals binding to cytoplasmic structures and a high affinity to proteins. It might happen that cytoplasmic proteins retain the main part of AV-153-Na molecules after exposure of the cells to the compound; only a small part of the molecules reaches DNA, where these activate DNA repair systems but do not produce harmful effects due to a very low local concentration in the nucleus.

Summarizing the data, it can be proposed that binding of the compound to DNA is identified by DNA repair systems as DNA lesions, and activity of DNA repair systems is increased. It seems that AV-153-Na *per se* does not induce mutations; however, it triggers the the activity of DNA repair enzymes, thus making cells less vulnerable by other mutagens.

## Conclusions

AV-153-Na protects the cells against genotoxic action of peroxynitrite although it does not react with peroxynitrite chemically. The protective effect is probably achieved due to direct interactions of the AV-153-Na with DNA and following activation of the DNA excision repair enzymes.

##  Supplemental Information

10.7717/peerj.4609/supp-1 Supplemental Information 1 Raw data of comet assay, 45 min incubation Primary results of comet assay of cells treated with peroxynitrite and AV-153 for 45 min, final results are given in Fig. 4.

10.7717/peerj.4609/supp-2 Supplemental Information 2 Raw data of comet assay, 3 h incubation Primary results of comet assay of cells treated with peroxynitrite and AV-153 for 3 h, final results are given in Fig. 4.

10.7717/peerj.4609/supp-3 Supplemental Information 3 Raw data of comet assay with AV-154-Na Primary results of comet assay of cells treated with peroxynitrite and AV-154, final results are given in Fig. 4.

10.7717/peerj.4609/supp-4 Supplemental Information 4 Raw data of ExSy SPOT assay, 0.1 mg of protein

10.7717/peerj.4609/supp-5 Supplemental Information 5 Raw data of GlycoSPOT assay, 1 mg of protein

10.7717/peerj.4609/supp-6 Supplemental Information 6 Raw data of GlycoSpot assay, 15 mg of protein

## Additional Information and Declarations

### Data Availability

The following information was supplied regarding data availability: The raw data are provided in the Supplemental File.
